# Echocardiographic Evaluation in Cardiac Resynchronization Therapy: A Single Center Experience

**DOI:** 10.7759/cureus.74344

**Published:** 2024-11-24

**Authors:** Jamilah S AlRahimi, Amjad A SaemAldahar, Anhar H Bahshwan, Joud G Alsulaimani, Yasser M Ismail, Ibrahim Jelaidan

**Affiliations:** 1 Cardiology, King Abdullah International Medical Research Center, King Abdulaziz Medical City, Ministry of National Guard Health Affairs, Jeddah, SAU; 2 College of Medicine, King Saud Bin Abdulaziz University for Health Sciences, Jeddah, SAU; 3 College of Applied Medical Sciences, King Saud Bin Abdulaziz University for Health Sciences, Jeddah, SAU; 4 Research, King Abdullah International Medical Research Center, King Abdulaziz Medical City, Ministry of National Guard Health Affairs, Jeddah, SAU; 5 Cardiology, King Abdulaziz Medical City, Ministry of National Guard Health Affairs, King Abdullah International Medical Research Center, Jeddah, SAU

**Keywords:** cardiac resynchronization therapy (crt), echocardiographic parameters, echocardiography, ejection fraction (ef), end-diastolic volume (edv), end-systolic volume (esv), heart failure, low left ventricular ejection fraction, transthoracic echocardiography (tte)

## Abstract

Introduction

Heart failure develops as a result of dysfunction in the cardiac muscle, which impairs the heart's ability to pump blood effectively. For this reason, many studies have shown that cardiac resynchronization therapy (CRT) has significantly reduced symptoms and improved cardiac function in patients with heart failure. Echocardiography is crucial in assessing CRT response, as it helps differentiate between patients who benefit from CRT and those who do not by evaluating key parameters like left ventricular ejection fraction (LVEF), a critical parameter in determining CRT eligibility. However, few studies focus specifically on the effectiveness of echocardiography for assessing CRT response, with existing research limited by a lack of standardized protocols and inadequate predictive tools. Accordingly, this study aims to assess the role of echocardiography in evaluating the efficacy of CRT in patients with heart failure at King Faisal Cardiac Center.

Methodology

This was a retrospective analytical cohort study that included all adult patients diagnosed with heart failure and underwent CRT between January 2017 and December 2021 at King Faisal Cardiac Center, King Abdulaziz Medical City, Jeddah, Saudi Arabia. Data were obtained from the Cardiac Non-invasive Lab, which was selected for its essential diagnostic tools for comprehensive echocardiographic evaluation of CRT efficacy. Study subjects were over 18 years old, diagnosed with heart failure with reduced ejection fraction (LVEF <35%), underwent CRT, and had echocardiograms at baseline and at least six months post-therapy. The collected data were retrieved from electronic medical records (BestCare; ezCaretech Co., Ltd,Seoul, South Korea), including relevant demographics and echocardiographic parameters such as end-systolic volume (ESV), end-diastolic volume (EDV), and ejection fraction (EF). Statistical analysis, paired t-tests, and Shapiro-Wilk test to assess data normality were conducted to evaluate pre- and post-CRT changes, with significance set at P<0.05.

Results

A total of 53 heart failure patients met the inclusion and exclusion criteria. The results of the study indicate statistically significant differences in the mean EF before and after CRT increased from 29.09±6.52% to 33.3±10.69% (p-value=0.0014). The mean ESV decreased from 114.46±60.63 mL to 97.13±65.89 mL, demonstrating a clinically significant improvement (p=0.056), and the mean EDV decreased from 157.08±64.67 mL to 138.87±78.07 mL (p = 0.0158). Furthermore, the EF increased by 14.47%, and the ESV decreased by 15.14% after CRT. These findings indicate improvement in left ventricular function following CRT.

Conclusion

The study demonstrates significant improvements in echocardiographic parameters based on echocardiogram findings, particularly the outcomes of EF and ESV after CRT in patients with heart failure with reduced ejection fraction. These findings highlight the potential of CRT as an effective therapy and aid in detecting responders to treatment. Nevertheless, the study is limited by a relatively small sample size, exclusion of comorbidities, and short follow-up period. Therefore, further longitudinal studies with larger cohorts and consideration of comorbidities are recommended.

## Introduction

Heart failure is a clinical syndrome characterized by impaired cardiac function due to anatomical and physiological weakness in the cardiac muscle (myocardium) followed by failure in filling and ejecting blood from the chambers, leading to inadequate circulation to meet the body's demands [1]. Furthermore, heart failure is a life-threatening disease that affects the cardiovascular system in adults with a prevalence of 2.5% in the United States, and it is demonstrated that heart failure has a high risk of death within one year for older adults [2,3]. Despite advancements in pharmacological therapies, including beta-blockers and angiotensin-converting enzyme (ACE) inhibitors, left ventricular systolic dysfunction remains a critical concern [2,4].

Cardiac resynchronization therapy (CRT) is now considered a standard treatment for heart failure, significantly decreasing morbidity and mortality and improving quality of life [2,5]. CRT works by improving the dyssynchronous heart with the capability to bring back the functional pattern and the contractility of the failing heart and is now considered one of the standard therapies for treating heart failure [5]. After the approval of CRT for 10 years, hundreds of thousands of patients have gone through the implantation of CRT devices globally [6]. Studies have also proven that CRT enhances left ventricle systolic and diastolic function, diminishes mitral regurgitation rate, improves New York Heart Association (NYHA) symptoms with a good prognosis by enhancing the functional capacity and quality of life, and reduces morbidity and mortality rates with the specific effectiveness in moderate to severe stages of heart failure [6-10]. In addition to the essential benefit on the left ventricle function, it also improves left and right atrial function associated with improvement of atrial compliance [6,11].

Echocardiography has been the most usable method in diagnosing cardiac desynchrony and CRT response, using techniques such as motion mode (M-mode) and tissue Doppler imaging (TDI) widely used in clinical practice [5]. The impact of CRT on cardiac structure and function is often evaluated using echocardiographic remodeling parameters, with a significant response defined as a reduction in left ventricle end-systolic volume (LVESV) by at least 15%, which predicts improved long-term survival and fewer hospitalizations [8,12-15]. Left ventricle ejection fraction (LVEF) is strongly related to prognosis, and its absolute increase of 5% or greater has been considered to be a positive response to CRT [16,17]. Many studies have used the LVESV and the LVEF as the major evaluating parameters to detect the significance of the CRT [15,16]. To illustrate, each echocardiographic variable primarily depends on the other because the equation to calculate the LVEF is as follows: (left ventricle end-diastolic volume (LVEDV)−LVESV)/LVEDV×100%, using the biplane Simpson method as an effective diagnostic tool to calculate the LVEF by automatically calculating each parameter after tracing the left ventricle and providing it in the report of each patient [7]. This highlights echocardiography's central role in providing precise and reliable measurements essential for evaluating CRT response, which can significantly improve patient selection and management strategies for CRT.

There are limited studies specifically examining the effectiveness of echocardiography in assessing CRT response, with current studies lacking standardized protocols and predictive tools for key parameters such as LVESV, LVEDV, and LVEF. Similarly, there is a lack of studies conducted in the Saudi Arabian population on the effectiveness of using echocardiography to assess the CRT response. Hence, this study aims to address these gaps by evaluating the role of echocardiography in assessing CRT response using remodeling parameters such as LVESV, LVEDV, and LVEF. The study was conducted at King Faisal Cardiac Center, Ministry of National Guard Health Affairs, Jeddah, Saudi Arabia, to contribute novel insights into the Saudi population’s CRT outcomes and improve patient management strategies.

## Materials and methods

This was a retrospective study conducted at the King Faisal Cardiac Center, King Abdulaziz Medical City, Ministry of National Guard Health Affairs - Western Region, Jeddah, Saudi Arabia, using Cardiac Non-invasive Lab data chosen for its crucial diagnostic tools for complete echocardiographic assessment of CRT efficacy. Existing clinical data was utilized to efficiently assess the long-term outcomes of CRT in all adult patients diagnosed with chronic heart failure with reduced ejection fraction LVEF<35% who underwent CRT between January 2017 and December 2021.

This study was conducted under ethical guidelines and was approved by the Institutional Review Board of King Abdullah International Medical Research Center (KAIMRC) (approval number: IRB/1858/22). To ensure the confidentiality and security of patient data, all records were anonymized by assigning a study code to each patient instead of their medical record numbers (MRN). Data were stored in a secure, password-protected electronic system, with access limited to authorized research personnel only.

Inclusion and exclusion criteria

Subjects were selected from heart failure clinics using a convenience-based consecutive sampling technique (nonprobability sampling). To minimize bias and enhance representativeness, we ensured that all eligible patients who received CRT within the study timeframe were considered for inclusion. Inclusion criteria were: age more than 18 years old, diagnosed with heart failure with reduced ejection fraction LVEF<35%, underwent CRT at least six months prior to the study period, and had an echocardiogram at baseline and one at least six months post therapy. We excluded patients who had lost follow-up or lacked echocardiographic data, applying the exclusion criteria consistently. Also, patients with multiple unrelated comorbidities or active medical conditions not associated with heart failure were excluded.

Sample size calculation

The power analysis indicated sufficient power for detecting meaningful differences in EF (0.92) but suggested that a larger sample size would be beneficial for a more robust analysis of ESV changes. The total selected list of patients was 89 but only 53 met our inclusion criteria.

Data collection

Data for this study were retrieved from the medical records using the electronic health information database system (BestCare; ezCaretech Co., Ltd, Seoul, South Korea), providing a reliable and standardized platform for retrieving patient demographic, clinical, and electrocardiographic data. The echocardiographic data was collected from the Xcelera Cardiology Information Management system (Koninklijke Philips N.V., Amsterdam, Netherlands) which has advanced capabilities in data collection and analysis, particularly for measuring LVESV, LVEDV, and LVEF, using the biplane Simpson method, a crucial tool for assessing CRT efficacy. The data were collected by the authors using a standardized data collection sheet to collect: (i) Demographics: age, gender, body surface area (BSA), (ii) Clinical characteristics: stroke volume (SV), left bundle branch block (LBBB), atrial fibrillation (A-Fib), (iii) Echocardiographic data: end-systolic volume (ESV), end-diastolic volume (EDV), ejection fraction (EF) pre and post-CRT.

To illustrate, in the patients' echocardiogram medical records, the ESV, EDV, and EF are assessed using the biplane Simpson method, which could be explained as tracing the left ventricle wall in the four-chamber view and the two-chamber view during systole and diastole. Then the machine would use the biplane Simpson’s equation and calculate all the measurements that are essential to evaluate the left ventricle.

Data analysis

The data were analyzed using JMP Pro 15 software (JMP Statistical Discovery LLC, Cary, North Carolina, United States), which offers advanced statistical analysis tools and visualizations suitable for evaluating clinical data that are ideal for this study. The data for continuous variables were present as mean and median. When the data were normally distributed, mean and standard deviation (SD) were used; otherwise, the median and interquartile range (IQR) were used when not normally distributed. The normality of data was assessed using the Shapiro-Wilk test. Data for categorical variables are presented as frequencies and percentages. Paired t-test was used to examine the association between EF and ESV before and after implanting the CRT device. A significance level of P-value less than 0.05 was considered statistically significant, as it is the conventional threshold in clinical research, balancing the risk of errors and the need for reliable results. No corrections for multiple comparisons were applied as the primary analysis focused on paired comparisons of pre- and post-CRT measurements. Figures and tables summarized demographic data, echocardiographic parameters, and comparative analyses of pre- and post-CRT measurements.

## Results

**Figure 1:**
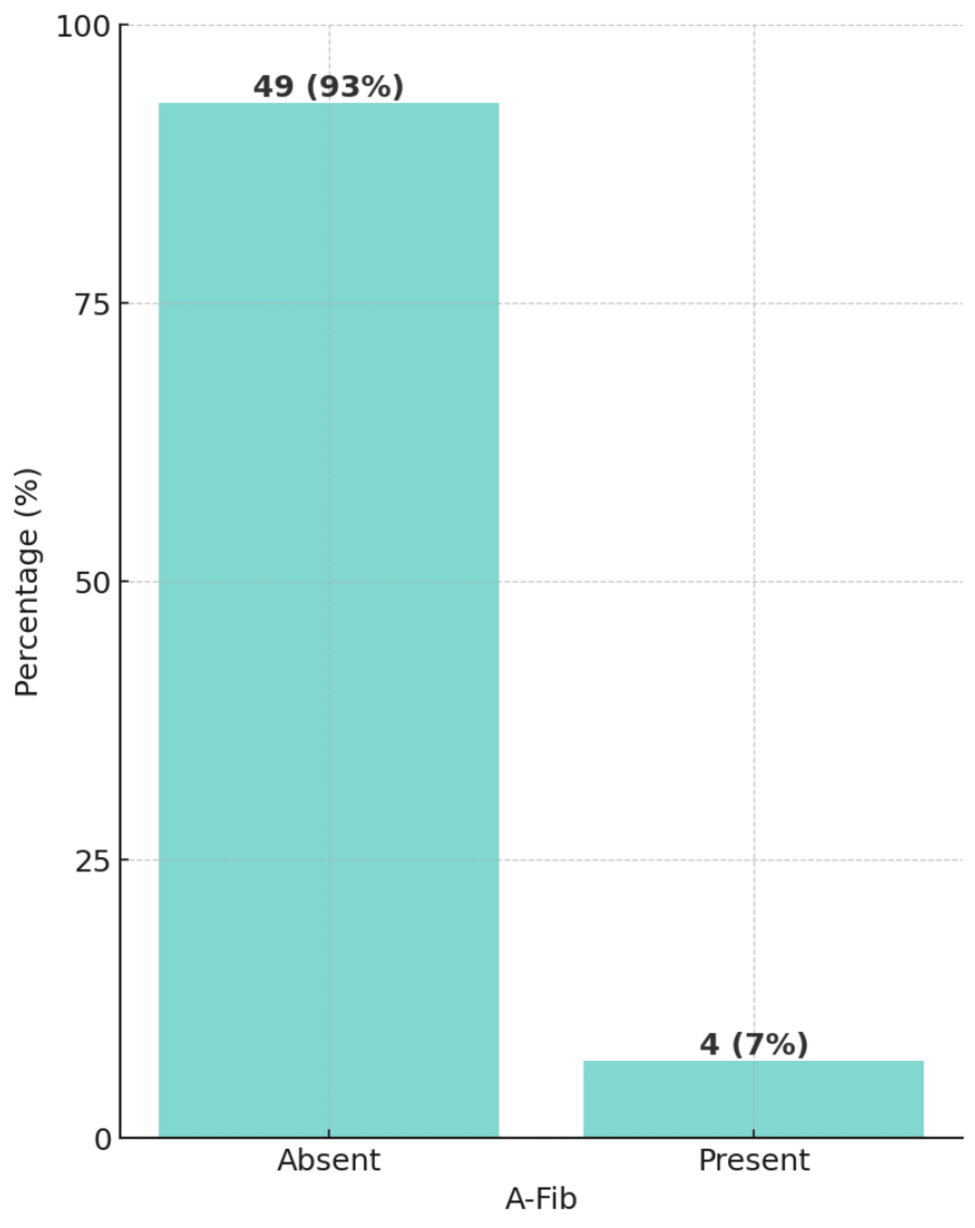
Bar diagram showing the distribution of atrial fibrillation (A-Fib) (N=53)

**Figure 2:**
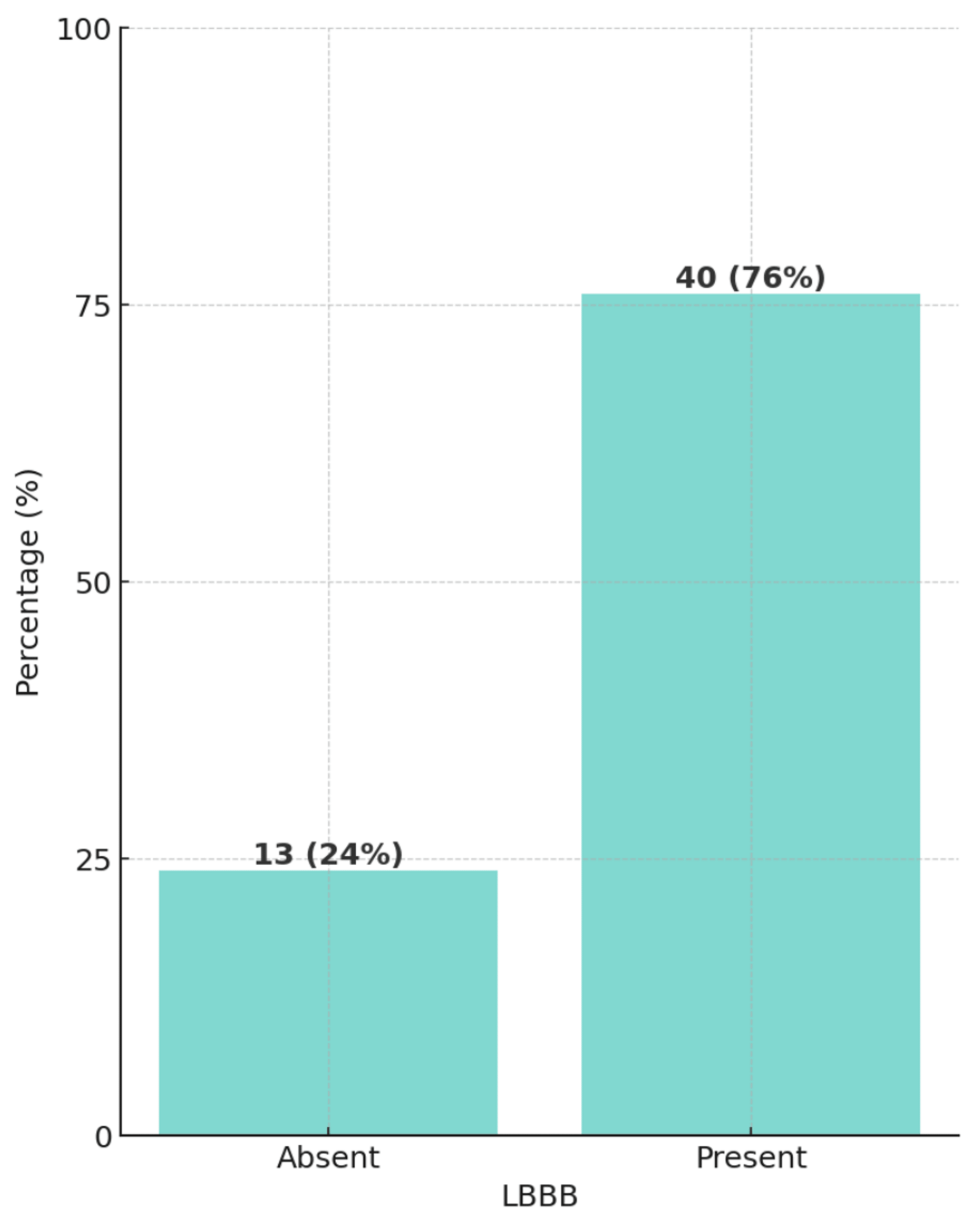
Bar diagram showing the distribution of left bundle branch block (LBBB) (N=53)

A total of 53 heart failure patients who went through CRT met the inclusion and exclusion criteria. Of the study population, 66% were male patients and 34% were female patients. Moreover, the clinical parameters of ECG showed a significant majority of the patients, accounting for 76%, were found to have LBBB (Figure 1). In contrast, only a small portion of the study population, 7%, was diagnosed with A-Fib (Figure 2). Demographics, clinical characteristics, and clinical parameters of ECG are summarized in Table 1.

**Table 1:** Descriptive statistics of demographics, clinical characteristics, and clinical parameters of ECG (N=53) IQR: interquartile range; BSA: body surface area; SV: stroke volume; LBBB: left bundle branch block; A-Fib: atrial fibrillation

Variable	Mean ± SD	Median, IQR	Frequency (Percentage)
Age (years)	65.5 ± 10.4	-	-
BSA (m²)	-	1.8, 0.31	-
SV (mL/beat)	53 ± 19	-	-
Gender (Male)	-	-	35 (66%)
Gender (Female)	-	-	18 (34%)
LBBB	-	-	40 (76%)
A-Fib	-	-	4 (7%)

**Figure 3:**
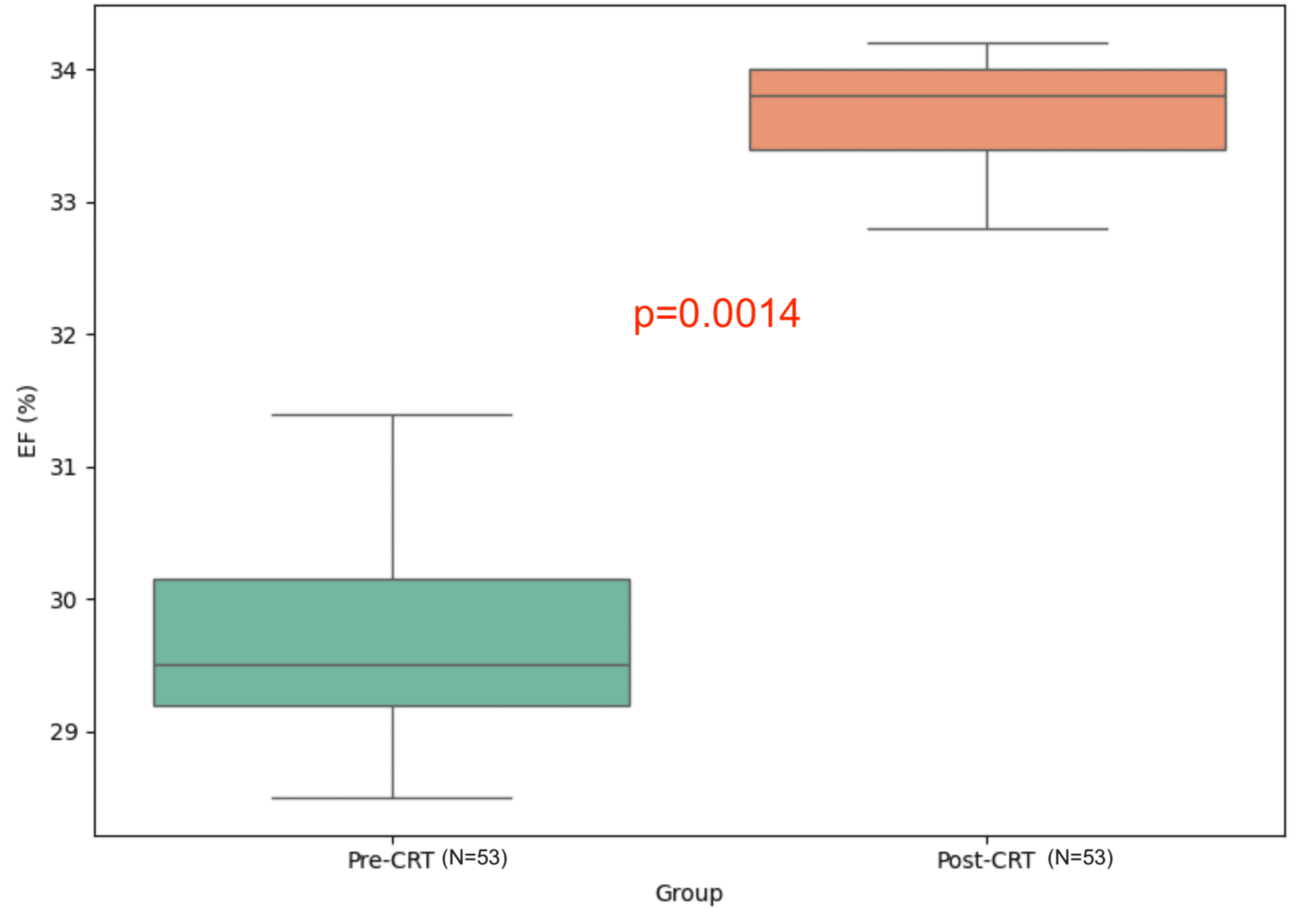
Box plot of ejection fraction (EF) before and after CRT (N=53) CRT: cardiac resynchronization therapy

**Figure 4:**
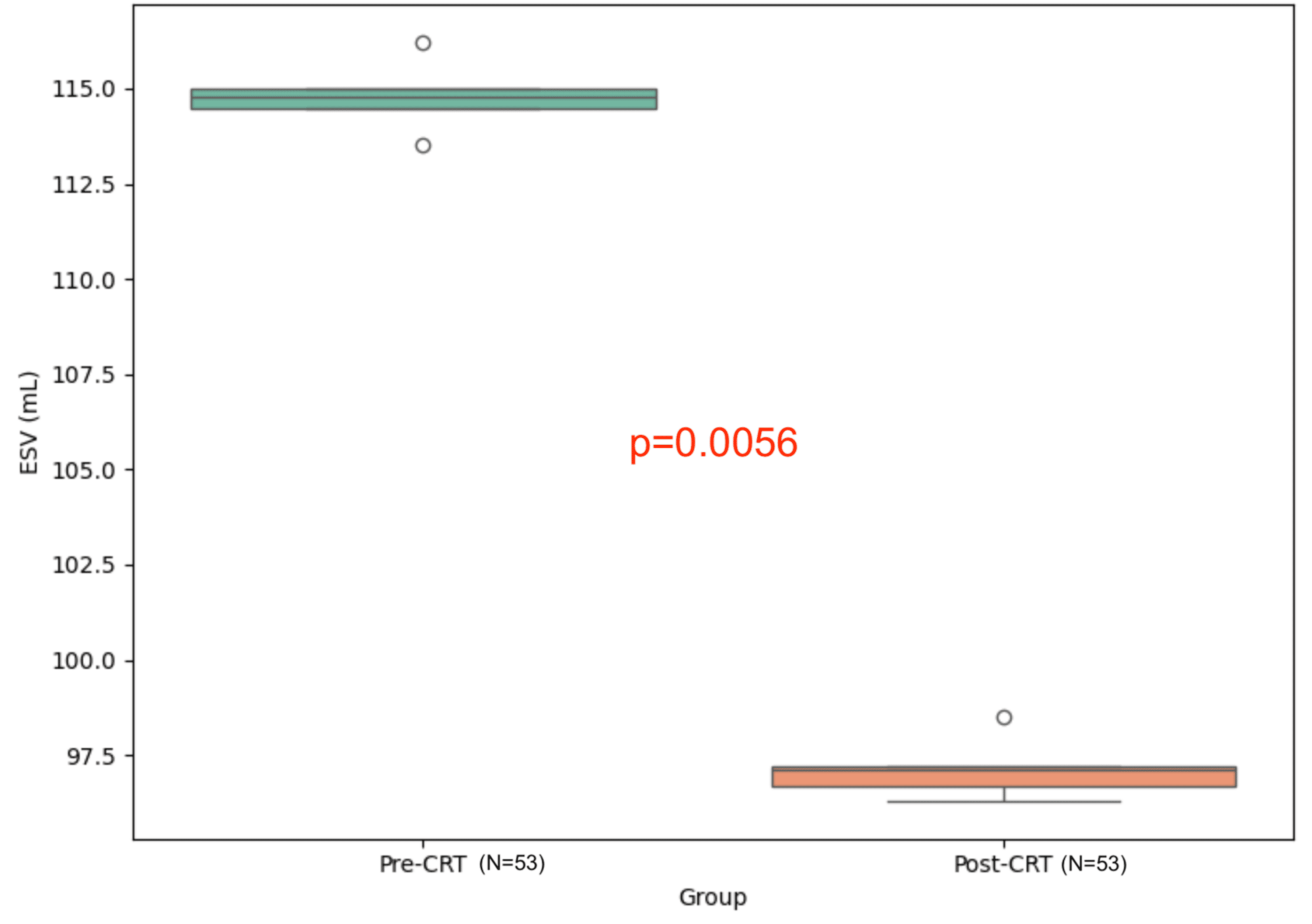
Box plot of left ventricle end-systolic volume (LVESV) before and after CRT (N=53) CRT: cardiac resynchronization therapy

Table 1 illustrates that the overall mean of patients’ age is 65.5 (SD±10.4) based on the Shapiro-Wilk test, which shows it is normally distributed. BSA is not normally distributed with a median of 1.8 and an IQR of 0.31. The SV has a normal distribution with a mean of 53 (SD±19). The results of the study indicate statistically significant differences in EF before and after implanting the CRT device as determined by paired t-test (p-value=0.0014) with a statistically significant increase in EF from a mean of 29.09% (SD ± 6.52) to 33.3% (SD ±10.69), representing an absolute increase of 4.21% (95% CI: 2.5-5.9) and a relative increase of 14.47%. Moreover, the ESV before and after the device implant showed clinical significance as well (p-value=0.0056), and it had significantly decreased from a mean of 114.46 mL (SD ± 60.63) to 97.13 mL (SD ±65.89), with an absolute reduction of 17.33 mL (95%CI: 11.2-23.5) and a relative reduction of 15.14% (Table 2, Figures 3, 4). Further, a multivariate correlation was assessed using Pearson’s correlation between EF before and after therapy, which showed a statistically significant positive correlation (r = 0.532, p-value = 0.0001). This moderate correlation suggests a meaningful association between pre- and post-therapy EF, with improvements in EF following CRT reflecting a positive and consistent effect. According to clinical guidelines, an improvement in EF of more than 5% or a reduction in ESV of 10% is considered clinically significant in heart failure management. The observed EF and ESV changes in this study are therefore within the range of clinically meaningful improvements.

**Table 2:** Mean difference between EF, ESV, and EDV before and after implanting the CRT device, statistical test used, and the p-value (N=53) EF: ejection fraction; ESV: end systolic volume; EDV: end diastolic volume: CRT: cardiac resynchronization therapy p value <0.05 is considered significant

Variable	Before or After CRT	Number of samples	Mean ± SD	Statistical test used	Test statistic	P value
EF (%)	Before	53	29.09 ± 6.52	Paired t-test	T= -4.226	0.0014
	After	53	33.3 ± 10.69			
ESV (mL)	Before	53	114.46 ± 60.63	Paired t-test	T= 17.330	0.0056
	After	53	97.13 ± 65.89			
EDV (mL)	Before	53	157.08 ± 64.67	Paired t-test	T= 18.212	0.0158
	After	53	138.87 ± 78.07			

## Discussion

Heart failure is reflected as a high mortality and morbidity disease due to the gradual deterioration of heart function and the reduction of EF, with significant impacts on quality of life and survival rates [2,5]. The analysis in the current study showed that CRT improved EF and reduced ESV, which led to significant improvements in left ventricular function. Therefore, these findings align with previous studies that highlight the role of CRT in improving key echocardiographic parameters, and reducing clinical symptoms associated with heart failure, showing a significant statistical association that exists between CRT and the echocardiographic parameters measured [2,9,16]. Accordingly, echocardiography plays an important role in evaluating desynchrony and it is the most common method for assessing EF and ESV [5].

While improvements in EF and reductions in ESV are central to assessing the effectiveness of CRT, it is crucial to understand how these changes translate into patient-centered outcomes. Several studies have shown that CRT leads to symptomatic improvement, better quality of life, and fewer hospitalizations, all of which are vital for assessing long-term benefits. For instance, Abraham et al. found that CRT implantation not only improved EF but also reduced hospitalization rates, providing evidence that improved heart function leads to better clinical outcomes [16]. In addition, Allison et al. [17] noted that CRT leads to significant symptomatic improvement in heart failure patients with LBBB, which was a characteristic of the majority of patients in this research. This results in better clinical benefit and a predictor of good outcomes as indicated in Maffessanti et al.'s study as well [15].

The mechanisms by which CRT improves EF and reduces ESV are believed to result from its ability to resynchronize left ventricular contractions, leading to improved left ventricular synchrony and more efficient blood pumping. Studies by Adamson et al. [4] and Serri et al. [5] have demonstrated that CRT improves heart rate variability and reduces dyssynchrony, which can result in better myocardial function and reduced ESV. Similarly, CRT may also influence other cardiac biomarkers, such as N-terminal pro b-type natriuretic peptide (NT-proBNP), which are indicative of heart failure severity and treatment response [15,18]. As highlighted by Yu et al.'s study, CRT can even reduce atrial dysfunction and improve atrial reverse remodeling, further contributing to its positive clinical outcomes [11].

In this study, the EDV, ESV, and EF are the main echocardiographic parameters used to assess the function of the heart. The increase in EF and the decrease in ESV in this study are consistent with findings from many other studies such as those by Bristow et al. [8] and Zhang et al. [9], who documented similar echocardiographic improvements following CRT implantation. Likewise, research conducted by Yu et al. [14] showed statistical significance in the same echocardiographic parameters that were also assessed by the biplane Simpson method in apical four-chamber and apical two-chamber views as well as Maffessanti et al.'s study [15]. The current study indicates a reduction of ESV by 15.1%. Hence, it is a remarkable outcome due to the importance of ESV, as it has been demonstrated in a study by White et al. that ESV is the most significant predictive factor of prognosis after recovery from myocardial infarction [13]. Therefore, the ESV and the echocardiographic parameters used in the study are considered most suitable to evaluate the heart after the recovery from heart failure and implantation of CRT.

Populations who suffer from heart failure are usually elderly and with a high risk of death at one year from diagnosis [3]. However, it is important to evaluate whether CRT benefits are consistent across different age groups. In our study, the average age of patients indicates that they are middle-aged or elderly, and the observed improvements in EF and reductions in ESV were consistent with findings in other studies involving predominantly elderly populations, such as Maffessanti et al. [15] and Allison et al. [17], who highlighted that CRT provides significant benefits even in the elderly. This aligns with the findings of Lindenfeld et al., who noted that while elderly patients do benefit from CRT, the extent of the benefit is influenced by comorbidities [18]. These results suggest that CRT's efficacy is not reduced by age itself but by the additional challenges of comorbidities and frailty. Furthermore, in this research, most of the patients were male, similar to a study by McKay et al. that aligns with CRT's efficacy in improving echocardiographic measures [19]. Thus, the significant reduction in ESV and increased EF in this cohort support the conclusion that CRT can result in meaningful improvements in heart function.

White et al. [13] and Yu et al. [14] demonstrated that a reduction in ESV by 10% predicts lower long-term mortality and fewer heart failure events. This highlights the role of echocardiographic parameters, specifically ESV in assessing long-term prognosis after CRT implantation. However, a study by Allison et al. argues that ESV and EF are not reliable predictors for morbidity and mortality [17]. The difference in outcomes between the current study and that of Allison et al. may arise from differences in sample demographics and measurement techniques. For instance, their study focused on a broader, older population, focusing more on frailty and comorbidities. It also included emerging measures such as the Global Longitudinal Strain (GLS), leading to contrasting results [17].

Future research should explore CRT's effectiveness in diverse subpopulations, and assess long-term follow-ups to evaluate sustained effects on mortality.

Strength and limitations

One of the strengths of this study is that the data were collected from a single center, which allowed for the use of the same standardized protocols for every patient. This consistency in data collection minimizes inter-center variability and ensures that the treatment protocols and diagnostic methods are standardized, which is essential for reducing confounding factors. Another key strength is the use of electronic health records for data collection. The implementation of electronic systems reduces the risk of data entry errors, such as transcription mistakes that are common with handwritten records, and enhances data accessibility. On the other hand, one of our limitations is the small sample size due to loss by follow-ups or not having preoperative echocardiographic studies. As a result, the final sample size may potentially limit the generalizability of the findings. Also, any other diseases or comorbidities that may worsen heart failures such as diabetes mellitus, kidney disease, and anemia have not been considered. Finally, the follow-up period may not have been sufficient to capture the long-term effects of CRT. Still, the echocardiographic results after CRT shown in this study are encouraging.

## Conclusions

The study showed that there are improvements in clinical parameters, particularly the outcomes of EF and ESV in patients with heart failure after the implantation of CRT. These findings suggest that CRT is effective in improving echocardiographic parameters and could potentially improve functional status and reduce hospitalization rates. However, further studies are recommended with additional aspects such as investigating CRT's effects in patients with specific comorbidities, analyzing predictors of CRT response, examining larger sample size, and longitudinal studies that would be valuable to assess the durability of CRT benefits over time, specifically regarding clinical outcomes like survival and hospitalization. The significant post-therapeutic echocardiographic findings are adequate to demonstrate that CRT is an effective therapy for patients with heart failure with reduced EF.

## References

[1] Inamdar AA, Inamdar AC (2016). Heart failure: diagnosis, management and utilization. J Clin Med.

[2] McAlister FA, Ezekowitz J, Hooton N (2007). Cardiac resynchronization therapy for patients with left ventricular systolic dysfunction: a systematic review. JAMA.

[3] Emmons-Bell S, Johnson C, Roth G (2022). Prevalence, incidence and survival of heart failure: a systematic review. Heart.

[4] Adamson PB, Kleckner KJ, VanHout WL, Srinivasan S, Abraham WT (2003). Cardiac resynchronization therapy improves heart rate variability in patients with symptomatic heart failure. Circulation.

[5] Serri K, Lafitte S, Amyot R, Sauvé C, Roudaut R (2007). Echocardiographic evaluation of cardiac dyssynchrony. Can J Cardiol.

[6] Daubert JC, Saxon L, Adamson PB (2012). 2012 EHRA/HRS expert consensus statement on cardiac resynchronization therapy in heart failure: implant and follow-up recommendations and management. Europace.

[7] St John Sutton MG, Plappert T, Abraham WT (2003). Effect of cardiac resynchronization therapy on left ventricular size and function in chronic heart failure. Circulation.

[8] Bristow MR, Gilbert EM, Abraham WT (1996). Carvedilol produces dose-related improvements in left ventricular function and survival in subjects with chronic heart failure. MOCHA Investigators. Circulation.

[9] Zhang Q, Fung JW, Auricchio A, Chan JY, Kum LC, Wu LW, Yu CM (2006). Differential change in left ventricular mass and regional wall thickness after cardiac resynchronization therapy for heart failure. Eur Heart J.

[10] Saba S, Marek J, Schwartzman D (2013). Echocardiography-guided left ventricular lead placement for cardiac resynchronization therapy: results of the Speckle Tracking Assisted Resynchronization Therapy for Electrode Region trial. Circ Heart Fail.

[11] Yu CM, Fang F, Zhang Q (2007). Improvement of atrial function and atrial reverse remodeling after cardiac resynchronization therapy for heart failure. J Am Coll Cardiol.

[12] Remme WJ (2001). The carvedilol and ACE-inhibitor remodelling mild heart failure evaluation trial (CARMEN)--rationale and design. Cardiovasc Drugs Ther.

[13] White HD, Norris RM, Brown MA, Brandt PW, Whitlock RM, Wild CJ (1987). Left ventricular end-systolic volume as the major determinant of survival after recovery from myocardial infarction. Circulation.

[14] Yu CM, Bleeker GB, Fung JW (2005). Left ventricular reverse remodeling but not clinical improvement predicts long-term survival after cardiac resynchronization therapy. Circulation.

[15] Maffessanti F, Jadczyk T, Wilczek J (2022). Electromechanical factors associated with favourable outcome in cardiac resynchronization therapy. Europace.

[16] Abraham WT, Fisher WG, Smith AL (2002). Cardiac resynchronization in chronic heart failure. N Engl J Med.

[17] Allison JD Jr, Biton Y, Mela T (2022). Determinants of response to cardiac resynchronization therapy. J Innov Card Rhythm Manag.

[18] Lindenfeld J, Powell BD, Hayes DL, Varma N, Jones P, Wold N, Saxon LA (2013). Mortality of patients with heart failure and reduced ejection fraction (HFrEF) who receive either ICD or CRT-D has improved yearly from 2003 to 2010: the Altitude Registry. J Card Fail.

[19] McKay B, Tseng NW, Sheikh HI (2021). Sex, race, and age differences of cardiovascular outcomes in cardiac resynchronization therapy RCTs: a systematic review and meta-analysis. CJC Open.

